# Cardiovascular and Gas Exchange Effects of Individualized Positive End-Expiratory Pressures in Cats Anesthetized With Isoflurane

**DOI:** 10.3389/fvets.2022.865673

**Published:** 2022-05-04

**Authors:** Marcela L. Machado, Joao H. N. Soares, Bruno H. Pypendop, Antonio J. A. Aguiar, Christina Braun, Gabriel C. Motta-Ribeiro, Frederico C. Jandre

**Affiliations:** ^1^William Pritchard Veterinary Medical Teaching Hospital, University of California, Davis, Davis, CA, United States; ^2^Department of Surgical and Radiological Sciences, School of Veterinary Medicine, University of California, Davis, Davis, CA, United States; ^3^Departamento de Cirurgia e Anestesiologia Veterinária, Faculdade de Medicina Veterinária e Zootecnia, UNESP—Univ. Estadual Paulista, Botucatu, Brazil; ^4^Anaesthesiology and Perioperative Intensive Care, University of Veterinary Medicine Vienna, Vienna, Austria; ^5^Laboratory of Pulmonary and Cardiovascular Engineering, Biomedical Engineering Program/COPPE, Federal University of Rio de Janeiro, Rio de Janeiro, Brazil; ^6^Biomedical Instrumentation Laboratory, Biomedical Engineering Program/COPPE, Federal University of Rio de Janeiro, Rio de Janeiro, Brazil

**Keywords:** mechanical ventilation, positive end-expiratory pressure (PEEP), cat, anesthesia, cardiovascular, gas exchange

## Abstract

**Objectives:**

To compare the effects of four levels of end-expiratory pressure [zero (ZEEP) and three levels of positive end-expiratory pressure (PEEP)] on the cardiovascular system and gas exchange of cats anesthetized with isoflurane and mechanically ventilated for 3 h with a tidal volume of 10 ml/kg.

**Study Design:**

Prospective, randomized, controlled trial.

**Animals:**

Six healthy male neutered purpose-bred cats.

**Methods:**

Anesthesia was induced with isoflurane and maintained at 1.3 minimum alveolar concentration. PEEP of maximal respiratory compliance (PEEP_maxCrs_) was identified in a decremental PEEP titration, and cats were randomly ventilated for 3 h with one of the following end-expiratory pressures: ZEEP, PEEP_maxCrs_ minus 2 cmH_2_O (PEEP_maxCrs−2_), PEEP_maxCrs_, and PEEP_maxCrs_ plus 2 cmH_2_O (PEEP_maxCrs+2_). Cardiovascular and gas exchange variables were recorded at 5, 30, 60, 120, and 180 min (T5 to T180, respectively) of ventilation and compared between and within ventilation treatments with mixed-model ANOVA followed by Dunnet's and Tukey's tests (normal distribution) or Friedman test followed by the Dunn's test (non-normal distribution). Significance to reject the null hypothesis was considered *p* < 0.05.

**Results:**

Mean arterial pressure (MAP—mmHg) was lower in PEEP_maxCrs+2_ [63 (49–69); median (range)] when compared to ZEEP [71 (67–113)] at T5 and stroke index (ml/beat/kg) was lower in PEEP_maxCrs+2_ (0.70 ± 0.20; mean ± SD) than in ZEEP (0.90 ± 0.20) at T60. Cardiac index, oxygen delivery index (DO_2_I), systemic vascular resistance index, and shunt fraction were not significantly different between treatments. The ratio between arterial partial pressure and inspired concentration of oxygen (PaO_2_/FIO_2_) was lower in ZEEP than in the PEEP treatments at various time points. At T180, DO_2_I was higher when compared to T5 in PEEP_maxCrs_. Dopamine was required to maintain MAP higher than 60 mmHg in one cat during PEEP_maxCrs_ and in three cats during PEEP_maxCrs+2_.

**Conclusion:**

In cats anesthetized with isoflurane and mechanically ventilated for 3 h, all levels of PEEP mildly improved gas exchange with no significant difference in DO_2_I when compared to ZEEP. The PEEP levels higher than PEEP_maxCrs−2_ caused more cardiovascular depression, and dopamine was an effective treatment. A temporal increase in DO_2_I was observed in the cats ventilated with PEEP_maxCrs_. The effects of these levels of PEEP on respiratory mechanics, ventilation-induced lung injury, as well as in obese and critically ill cats deserve future investigation for a better understanding of the clinical use of PEEP in this species.

## Introduction

The application of positive end-expiratory pressure (PEEP) and alveolar recruitment maneuvers (ARM) during mechanical ventilation can increase functional residual capacity (FRC) and reduce or treat small airway closure and atelectasis in humans (1, 2) and dogs (3). Despite an improvement in arterial oxygenation related to PEEP in dogs (3), the same effect was not observed in other studies using the same species (4, 5). To the authors' best knowledge, no study on the effects of PEEP on gas exchange, cardiac output, and oxygen delivery in cats has been published. However, PEEP may have a beneficial effect on gas exchange in this species because atelectasis has been observed when anesthetized cats were ventilated with zero PEEP (ZEEP) (6, 7). Despite its potential benefits of improving FRC and gas exchange, PEEP can result in decreased mean arterial pressure (MAP) in cats (7). The cardiovascular depression caused by PEEP is mainly related to a decrease in cardiac index (CI) (8) as reported in dogs (5).

The PEEP of maximal respiratory system compliance (PEEP_maxCrs_) achieved during a decremental PEEP titration has been recently used as a method to individualize PEEP in protocols of protective ventilation (9, 10). In healthy rats, PEEP_maxCrs_ promoted a better balance between alveolar overdistention and tidal recruitment/derecruitment when compared to higher PEEP or the absence of it (11). In addition, PEEP levels higher than PEEP_maxCrs_ seems to provide better prevention of atelectasis but at the expense of causing alveolar overdistension (11, 12) impairing cardiovascular function (5), and possibly causing redistribution of pulmonary blood with an increase in ventilation/perfusion ($\overset{∙}{\text{V}}$/$\overset{∙}{\text{Q}}$) mismatch (13).

This study aimed to compare the effects of four levels on end-expiratory pressure (EEP): ZEEP, PEEP_maxCrs_ minus 2 cmH_2_O (PEEP_maxCrs−2_), PEEP_maxCrs_, and PEEP_maxCrs_ plus 2 cmH_2_O (PEEP_maxCrs+2_) on the cardiovascular system, pulmonary gas exchange, and arterial oxygenation in isoflurane-anesthetized cats. We hypothesized that in isoflurane-anesthetized cats with healthy lungs mechanically ventilated for 3 h: (1) PEEP_maxCrs_ and PEEP_maxCrs+2_ will provide higher arterial oxygenation than ZEEP and PEEP_maxCrs−2_; and (2) PEEP_maxCrs+2_ will decrease CI, MAP, and oxygen delivery index (DO_2_I) when compared to ZEEP.

## Materials and Methods

### Animals

Six healthy male neutered cats, 1–2 years old, weighing 5.1 ± 0.9 kg (mean ± standard deviation) were enrolled in this prospective, randomized, controlled crossover study. All 6 cats received all EEP treatments in separate days with a minimum of 7 days between experiments. Physical examination and routine basic blood work (packed cell volume and serum biochemistry) were performed to evaluate the cats' health status. All cats were housed in a room at the Teaching and Research Animal Care Services facility, University of California, Davis, United States. All cats were acclimatized to the laboratory conditions and handled 14 days before commencing the study. Cats were fed a commercial diet once a day and had access to water *ad libitum*. This study was approved by the Institutional Animal Care and Use Committee of the University of California Davis (n. 21985). Food, but not water, was withheld for 12 h before the experiments.

### Instrumentation

**Figure 1:**
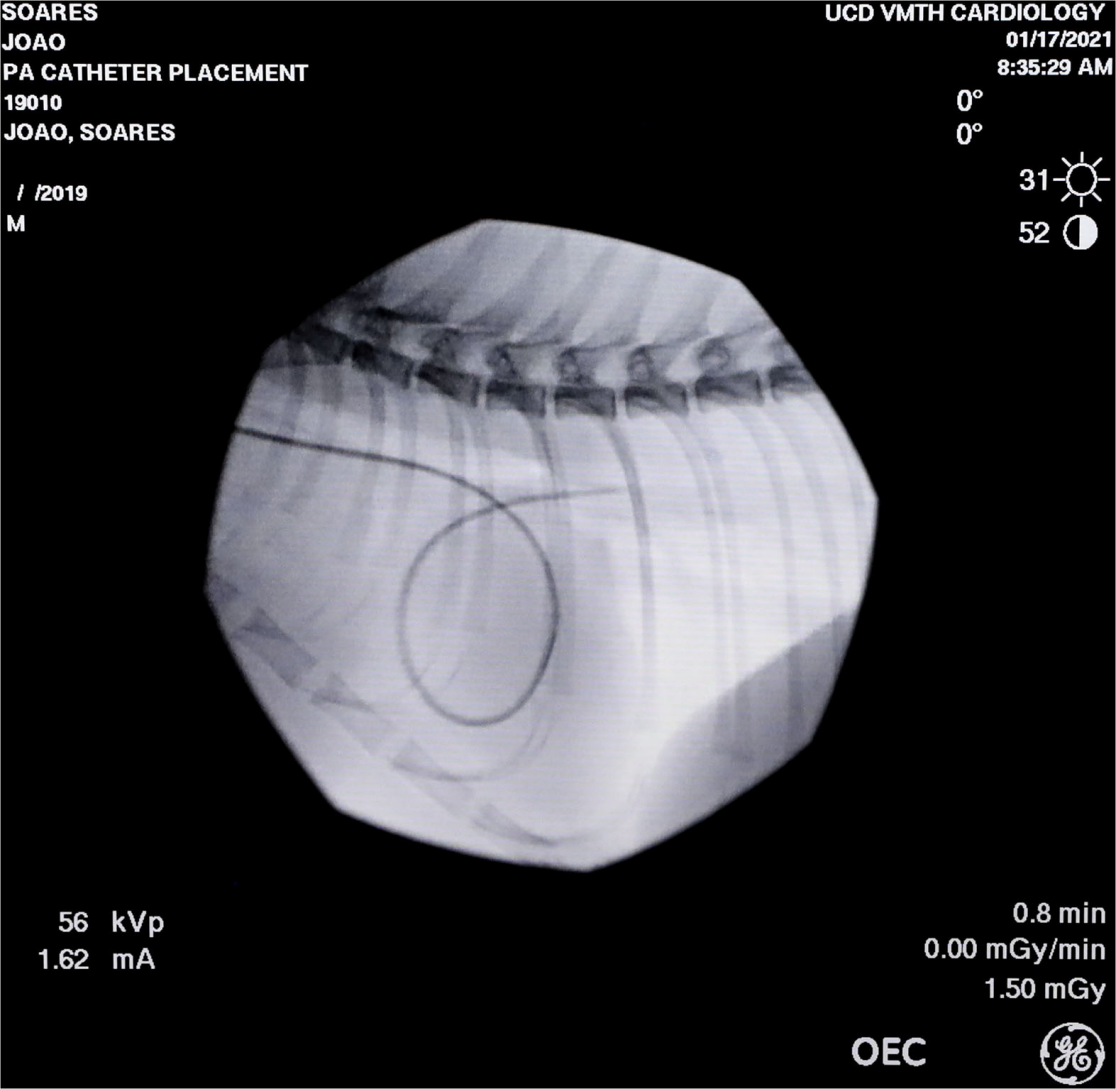
Fluoroscopic image of the thermodilution catheter positioned at the pulmonary artery of one of the studied cats.

Each cat was anesthetized with 5% isoflurane (Isoflurane; Piramal Critical Care Inc., PA, USA) in oxygen (5 L/min) delivered by a Bain circuit into an acrylic chamber. Once the righting reflex was lost, the trachea was intubated with a 4.5-mm internal diameter cuffed tube (Sheridan/CF, Teleflex, NC, USA), and anesthesia was maintained during the whole experiment with 1.3 MAC of isoflurane (2.12 ET_ISO_%) (14) in oxygen delivered from a circle breathing system. A 22-gauge catheter (BD Insyte, 2.5 cm, USA) was aseptically inserted in a cephalic vein for infusion of lactated Ringer's solution (Baxter Healthcare Corp., IL, USA) at 3 ml/kg/h. A pulse oximetry probe was positioned on the tongue and a lead II electrocardiogram was connected to evaluate heart rhythm. The cats were placed in dorsal recumbency during the whole experiment and rocuronium bromide (XGen Pharmaceuticals DJB, IL, USA) at a dose of 0.6 mg/kg was administered intravenously followed by a constant rate infusion of 0.6 mg/kg/h. If any sign of spontaneous ventilation was observed on the monitoring of airway pressure (*P*_*aw*_) or flow ($\overset{∙}{V}$), an additional dose of 0.1 mg/kg of rocuronium was administered intravenously. Stimulating electrodes were placed over the peroneal nerve and an accelerometer was attached to the paw to monitor the train-of-four ratio (TOF-Watch SX, Organon Ltd., Ireland). The train-of-four ratio was maintained below 0.3 during the whole experiment. Mechanical ventilation was performed during instrumentation in volume-control mode with a tidal volume (V_T_) of 10 mL/kg, inspiratory-to-expiratory time ratio (I:E ratio) of 1:2, FIO_2_ between 0.95 and 0.98, and the respiratory rate (*f*_*R*_) adjusted to maintain the end-tidal CO_2_ partial pressure (PETCO_2_) between 30 and 40 mmHg (baseline ventilatory settings) (Flow-I, C20, Getinge AB, USA). Inspired fraction of oxygen (FIO_2_) and end-tidal isoflurane concentration (ET_ISO_) were measured with a gas analyzer calibrated before and during the experiments (AS/3, Datex-Ohmeda, Helsinki, Finland). A mainstream neonatal capnography sensor (NM3, Philips Healthcare, MA, USA) was placed between the pneumotachometer and the breathing system to measure PETCO_2_. Calibration curves for isoflurane, oxygen, and CO_2_ concentrations were obtained by linear regression using 3 different concentrations of primary gas standards (1.33, 2.00, and 3.50% for isoflurane; 60, 80, and 100% for O_2_; 5.0, 8.1, and 10.0% for CO_2_). A 4-Fr 5.5 cm sheath introducer (RCFN-4.0-18-5.5-RA1.5; Cook Medical, IN, USA) was aseptically inserted into the jugular vein using a modified Seldinger technique and sutured to the skin. A 4-Fr, 75 cm thermodilution catheter (AI-07044; Teleflex, NC, USA) was placed through the introducer until its tip was positioned in the pulmonary artery. Positioning was confirmed by fluoroscopy (Figure 1) with further adjustments in position made based on visualization of the pulmonary artery and central venous pressure (CVP) waveforms and the ability of the catheter to occlude the pulmonary artery during inflation of its balloon. The femoral or carotid artery was aseptically catheterized with a 22-gauge, 8 cm catheter (Arteriofix V, B Braun Meslsungen AG, Germany). Pressure transducers (Meritrans DTX plus, MeritMedical, Singapore) connected to non-compliant tubing filled with heparinized saline (2 U/ml) were positioned and zeroed at the level of the scapulohumeral joint, and attached to the arterial catheter, and the proximal and distal ports of the thermodilution catheter, for the recordings of MAP, CVP, and mean pulmonary artery (MPAP) pressure, respectively. Pressure transducers were calibrated against a mercury or water column before each experiment and connected to a data acquisition system as previously described (5). The dampening coefficient and natural frequency response of the blood pressure transducers-catheter were assessed at the beginning of the experiments by the fast flush test response, consisting of a quick opening of the flush valve of the transducer to the pressurized bag (~300 mmHg) with heparinized saline. The typical dampening coefficient of the system and natural frequency response were 0.3 (0.25 to 0.4) and 15 Hz (12 to 25 Hz), respectively. The thermodilution catheter thermistor was connected to a cardiac output monitor (AS/3, Datex-Ohmeda, Helsinki, Finland). Body temperature was measured by the thermistor of the thermodilution catheter and was maintained between 37.5 and 38.5°C by heating blankets. Thermodilution cardiac output was measured by the fast injection (1–2 s) of 1.5 ml of cold saline (0–2°C) through the proximal lumen of the pulmonary artery thermodilution catheter and the average of three measurements with <10% difference was reported.

A Lilly heated pneumotachometer (8300 series; Hans Rudolph Ltd., KS, USA) coupled to a differential pressure transducer (DPL2.5—Hugo Sacks Elektronik—Harvard Apparatus GmbH, Germany) was connected between the breathing system and the endotracheal tube for the measurements of airflow ($\overset{∙}{V}$). Airway pressure (*P*_aw_) was measured by a differential pressure transducer (MPX 399/2, Hugo Sacks Elektronik—Harvard Apparatus GmbH, Germany) from a port between the endotracheal tube and the pneumotachometer. Volume (*V*) was calculated by the numerical integration of $\overset{∙}{V}$, zeroed at the beginning of each cycle. An esophageal balloon catheter was placed in the esophagus for the measurements of esophageal pressure (*P*_eso_) (P75, Hugo Sacks Elektronik—Harvard Apparatus GmbH, Germany) as a surrogate of pleural pressure. The position of the esophageal balloon catheter was verified by the occlusion method (15). The digital signals of *P*_aw_, $\overset{∙}{V}$, and *P*_eso_ were continuously acquired at 400 Hz and displayed by a custom-made software (16) written in LabView (LabView 2019, NI, TX, USA), and saved on a personal computer. Values of *P*_aw_ and *P*_eso_ were calibrated by linear regression using 6 pressure points (−10, 0, 10, 20, 30, and 40 cmH_2_O) provided by a water column (reference method). The calibration of $\overset{∙}{V}$ was performed by a modified flow-integration method (5, 17) using a 100-ml volumetric calibration syringe (5510 series—Hans Rudolph Ltd., KS, USA) containing oxygen and isoflurane concentrations similar to those used during the experiments (O_2_ 94–99%; isoflurane 2.0–2.2%).

### Experimental Protocol

After instrumentation, an intravenous bolus of 10 ml/kg of lactated Ringer's solution was administered over 5 min. Then, an ARM was performed in pressure-control ventilation with 10 cmH_2_O difference between peak airway pressure and EEP in 4 ascending steps of EEP (0, 5, 10, 15, and 20 cmH_2_O), with each step maintained for 30 s. Following this ARM, a descendent PEEP titration from 10 to 0 cmH_2_O in steps of 2 cmH_2_O, maintained for 2 min each was performed in volume-control mode with *V*_*T*_ of 10 ml/kg and the same *f*_R_ used in the baseline ventilatory settings.

Immediately after the PEEP titration, respiratory system compliance (*C*_rs_) at each PEEP step was estimated offline using custom-made software (18) written in MATLab (MathWorks Inc., MA, USA). For this purpose, the multiple linear regression method was applied to the single compartment equation of motion of the respiratory system presented below:


$$\begin{array}{l} P_{\text{aw}}{\left(t\right)=\overset{∙}{V}\left(t\right)\times R}_{\text{rs}}{+V\left(t\right)\times E}_{\text{rs}}{+P}_{0}, \end{array}$$

where *R*_rs_ and *E*_rs_ are respiratory system resistance and elastance, respectively, *P*_0_ is the *P*_aw_ when *V* and $\overset{∙}{V}$ are zero, and *t* is time. Respiratory system compliance (*C*_rs_) was calculated as 1/*E*_rs_.

The PEEP step associated with the highest *C*_rs_ was assigned as PEEP_maxCrs_, as previously described (5). After the PEEP titration, a second ARM identical to the first one was performed and the cats were mechanically ventilated for 3 h with one of the randomized (www.randomizer.org) EEP treatments (ZEEP, PEEP_maxCrs−2_, PEEP_maxCrs_, and PEEP_maxCrs+2_). An intravenous constant rate infusion of dopamine, starting at 5 mcg/kg/min and increased as needed, was used to maintain MAP higher than 60 mmHg. Mechanical ventilation was performed in volume-control mode, *V*_*T*_ of 10 mL/kg, I:E ratio of 1:2, FIO_2_ between 0.95 and 0.98, no inspiratory pause, an inspiratory rise time of 0%, and *f*_R_ was adjusted to maintain PETCO_2_ between 28 and 35 mmHg. The cardiovascular and gas exchange data were collected at 5, 30, 60, 120, and 180 min (T5–T180, respectively) of mechanical ventilation with the investigated EEP. Arterial and mixed venous blood samples (1 ml) were simultaneously and anaerobically collected for the immediate measurement of their respective hemoglobin concentration (Hba and Hb$\overset{\bar{}}{v}$), hemoglobin oxygen saturation (SaO_2_ and S$\overset{\bar{}}{v}$O_2_) (OSM 3 co-oximeter, Radiometer, CA, USA), partial pressure of carbon dioxide (PaCO_2_ and P$\overset{\bar{}}{v}$CO_2_), partial pressure of oxygen (PaO_2_ and P$\overset{\bar{}}{v}$O_2_), lactate concentration, and pH (pHa and pH$\overset{\bar{}}{v}$) (ABL825, Radiometer Medical ApS, Denmark). The blood gas values were corrected to the actual body temperature at the time of blood collection. Once the train-of-four ratio had been ≥100 % for more than 15 min and the cats resumed spontaneous ventilation, a bronchoalveolar lavage unrelated to this study was performed with 10 ml of saline (37°C) in the right caudal lung lobe under bronchoscopy guidance. After that, the delivery of isoflurane was stopped, and the cats were recovered from anesthesia. Meloxicam (VetOne, ID, USA) at a dose of 0.2 mg/kg was administered subcutaneously at the end of the experiment, and cats were returned to the vivarium. After the completion of the study, all cats were adopted to individuals pre-selected by the University of California, Davis IACUC.

Heart rate (HR), systolic (SAP), MAP, and diastolic (DAP) arterial pressures, CVP, MPAP, pulmonary artery occlusion pressure (PAOP), cardiac output (CO), *f*_*R*_, PETCO_2_, ET_ISO_, and body temperature were measured during the experiments. Stroke index (SI), CI, systemic vascular resistance index (SVRI), pulmonary vascular resistance index (PVRI), arterial blood oxygen concentration (CaO_2_), mixed venous blood oxygen concentration (C$\overset{\bar{}}{v}$O_2_), oxygen delivery index (DO_2_I), oxygen consumption index (VO_2_I), oxygen extraction ratio (O_2_ER), PaO_2_:FIO_2_, shunt fraction (Qs/Qt), and PaCO_2_ minus PETCO_2_ [P(a-ET)CO_2_] were calculated using standard formulae (19, 20).

### Statistical Analysis

The primary outcomes of the study were HR, MAP, CI, SI, MPAP, CVP, SVRI, PVRI, Qs/Qt, and DO_2_I. Because this is the very first study evaluating the cardiovascular effects of PEEP in cats, there was no previous data set to perform an optimal power analysis. Alternatively, cardiovascular data from a previous study performed in cats from the same colony and in similar laboratory conditions were used (21). Six cats were enough to detect a difference of 20–30% (effect size of 1.1) in the primary outcomes of this project between and within each EEP treatment with a power of 0.8151 and an alpha level of 0.05. The numeric data were verified for normality with the Shapiro–Wilk test. Normally and non-normally distributed data are reported as mean ± standard deviation (SD) and median (range), respectively. A Kruskal–Wallis test followed by Dunn's test was performed to compare the PEEP_maxCrs_ identified for each EEP treatment. All cardiovascular and respiratory variables were compared between treatments and within each treatment. For normally distributed data, a mixed model analysis of variance (using EEP treatment, timepoints, and their interaction as fixed effects and cat as a random effect) followed by Dunnett's test to compare each time point with T5 within each treatment, and Tukey's procedure for comparisons between treatments within the same time point were used. For non-normally distributed data, the Friedman test followed by Dunn's test for comparisons between each time point and T5 within the same treatment, and for comparisons between treatments within the same time point. The level of significance for all statistical analyses was *p* ≤ 0.05.

## Results

All cats recovered from all anesthetic episodes without complications. Data from one cat in PEEP_maxCrs_ at T180 were not included in the results due to arterial catheter malfunction during that time point. The order of treatment administration in the cats of the study is presented in Table 1.

**Table 1:** Order of end-expiratory treatments in the six cats of this study.

**Cat**	**Treatment 1**	**Treatment 2**	**Treatment 3**	**Treatment 4**
1	PEEP_maxCrs−2_	PEEP_maxCrs_	PEEP_maxCrs+2_	ZEEP
2	PEEP_maxCrs+2_	ZEEP	PEEP_maxCrs_	PEEP_maxCrs−2_
3	PEEP_maxCrs_	PEEP_maxCrs−2_	ZEEP	PEEP_maxCrs+2_
4	PEEP_maxCrs+2_	ZEEP	PEEP_maxCrs−2_	PEEP_maxCrs_
5	PEEP_maxCrs+2_	PEEP_maxCrs_	PEEP_maxCrs−2_	ZEEP
6	PEEP_maxCrs−2_	PEEP_maxCrs+2_	ZEEP	PEEP_maxCrs_

*ZEEP, zero end-expiratory pressure, PEEP_maxCrs_, positive end-expiratory pressure of highest respiratory system compliance, PEEP_maxCrs__−2_,PEEP_maxCrs_ minus 2 cmH_2_O; and PEEP_maxCrs+_2, PEEP_maxCrs_ plus 2 cmH_2_O*.

PEEP_maxCrs_ was 4 (4–4), 4 (4–6), 4 (4–4), and 4 (4–6) cmH_2_O in ZEEP, PEEP_maxCrs−2_, PEEP_maxCrs_, and PEEP_maxCrs+2_, respectively, with no significant difference between treatments. The values of body temperature, ET_ISO_, V_T_, *f* , and EEP measured during the experiments are presented in Table 2. No significant differences between and within each EEP treatment were observed for body temperature, ET_ISO_, and V_T_, while EEP was significantly different between EEP treatments at all time points (*p* < 0.0001), as expected from the study design.

**Table 2:** Temperature, end-tidal isoflurane concentration, and ventilator variables in six isoflurane-anesthetized cats mechanically ventilated for 3 h with a tidal volume of 10 ml/kg and four end-expiratory pressure (EEP) treatments: zero end-expiratory pressure (ZEEP), positive end-expiratory pressure (PEEP) of highest respiratory system compliance (PEEP_maxCrs_), PEEP_maxCrs_ minus 2 cmH_2_O (PEEP_maxCrs−2_), and PEEP_maxCrs_ plus 2 cmH_2_O (PEEP_maxCrs+2_).

**Variable**	**Group**	**T5**	**T30**	**T60**	**T120**	**T180**
Temp	ZEEP	38.4 ± 0.4	38.6 ± 0.2	38.6 ± 0.2	38.3 ± 0.3	38.3 ± 0.2
(°C)	PEEP_maxCrs−2_	38.4 ± 0.4	38.3 ± 0.4	38.2 ± 0.4	38.4 ± 0.3	38.0 ± 0.4
	PEEP_maxCrs_	38.4 ± 0.3	38.4 ± 0.2	38.4 ± 0.3	38.5 ± 0.4	38.4 ± 0.4
	PEEP_maxCrs+2_	38.5 ± 0.3	38.5 ± 0.2	38.5 ± 0.3	38.3 ± 0.3	38.6 ± 0.4
ET_ISO_	ZEEP	2.0 (1.9–2.1)	2.0 (1.9–2.1)	2.1 (1.9–2.2)	2.1 (1.9–2.2)	2.1 (1.9–2.1)
(%)	PEEP_maxCrs−2_	2.2 (2.1–2.4)	2.2 (2.1–2.4)	2.2 (2.1–2.4)	2.3 (2.1–2.5)	2.2 (2.1–2.4)
	PEEP_maxCrs_	2.1 (1.9–2.2)	2.2 (2.1–2.3)	2.2 (2.0–2.3)	2.1 (2.0–2.3)	2.1 (2.0–2.2)
	PEEP_maxCrs+2_	2.1 (2.0–2.4)	2.1 (1.9–2.4)	2.2 (2.0–2.4)	2.1 (2.0–2.4)	2.2 (2.0–2.5)
V_T_	ZEEP	9.6 ± 0.4	9.6 ± 0.4	9.6 ± 0.4	9.7 ± 0.4	9.7 ± 0.4
(mL/kg)	PEEP_maxCrs−2_	9.7 ± 0.5	9.7 ± 0.5	9.7 ± 0.5	9.8 ± 0.4	9.8 ± 0.4
	PEEP_maxCrs_	9.7 ± 0.2	9.8 ± 0.2	9.9 ± 0.2	10.0 ± 0.2	10.0 ± 0.2
	PEEP_maxCrs+2_	10.0 ± 0.7	9.9 ± 0.2	10.0 ± 0.2	9.9 ± 0.2	10.0 ± 0.2
PIP	ZEEP	5.1 ± 0.4^A^	5.7 ± 0.5^A^	6.1 ± 0.4^*^^A^	6.5 ± 0.5^*^^A^	6.8 ± 0.6^*^^A^
(cmH_2_O)	PEEP_maxCrs−2_	7.4 ± 1.2^B^	7.9 ± 1.1^B^	8.1 ± 1.1^B^	8.4 ± 1.2^*^^B^	8.9 ± 1.4^*^^B^
	PEEP_maxCrs_	8.5 ± 1.1^C^	9.0 ± 1.3^B^	9.2 ± 1.0^B^	9.7 ± 1.2^B^	10.0 ± 1.2^*^^B^
	PEEP_maxCrs+2_	11.6 ± 1.6^D^	12.1 ± 1.7^C^	12.4 ± 1.7^C^	13.0 ± 2.2^C^	13.0 ± 2.2^C^
*f_*R*_* (breath/min)	ZEEP	26 ± 2	25 ± 3	24 ± 3	24 ± 2	24 ± 3
	PEEP_maxCrs−2_	26 ± 3	26 ± 3	26 ± 3	25 ± 2	25 ± 2
	PEEP_maxCrs_	25 ± 3	26 ± 3	26 ± 3	26 ± 3	26 ± 3
	PEEP_maxCrs+2_	24 ± 2	26 ± 4	26 ± 4	27 ± 2	27 ± 2
EEP	ZEEP	0.2 ± 0^A^	0.2 ± 0^A^	0.1 ± 0.1^A^	0.1 ± 0.1^A^	0.2 ± 0^A^
(cmH_2_O)	PEEP_maxCrs−2_	2.7 ± 0.8^B^	2.7 ± 0.8^B^	2.8 ± 0.9^B^	2.7 ± 0.8^B^	2.7 ± 0.8^B^
	PEEP_maxCrs_	4.2 ± 0.9^C^	4.2 ± 0.8^C^	4.2 ± 0.7^C^	4.2 ± 0.9^C^	4.2 ± 0.9^C^
	PEEP_maxCrs+2_	6.3 ± 1.0^D^	6.5 ± 0.9^D^	6.6 ± 0.9^D^	6.5 ± 0.9^D^	6.4 ± 0.8^D^

*Data are presented as mean ± standard deviation or median (range)*.

*Temp, temperature; ET_ISO_, end-tidal isoflurane concentration; V_T_, tidal volume; f_R_, respiratory rate*.

*Different superscript letters within each timepoint indicate a significant difference between treatments*.

*Different letters within a column indicate significant difference*.

* *Significant different from T5*.

The cardiovascular results for each treatment are summarized in Table 3. MAP was significantly lower at T5 during PEEP_maxCrs+2_ compared to ZEEP (*p* = 0.0492). At T60, SI was significantly lower during PEEP_maxCrs+2_ compared to ZEEP (*p* = 0.0135). MPAP was significantly higher during PEEP _maxCrs+2_ compared to ZEEP at all timepoints (T5 *p* = 0.0107; T30 *p* = 0.002; T60, T120 and T180 *p* < 0.0001), when compared to PEEP_maxCrs−2_ at T60 (*p* = 0.0209), T120 (*p* = 0.0149) and T180 (*p* = 0.0147), and when compared to PEEP_maxCrs_ at T60 (*p* = 0.0252). CVP was significantly lower during ZEEP compared to PEEP_maxCrs+2_ at T5 (*p* = 0.0344) and T30 (*p* = 0.0292). PVRI was higher during PEEP_maxCrs_ compared to ZEEP at T30 (*p* = 0.0131), T120 (*p* = 0.036), and T180 (*p* =0.0202), and compared to PEEP_maxCrs−2_ at T30 (*p* = 0.0202) and T180 (*p* = 0.0306). PVRI was also higher during PEEP_maxCrs+2_ compared to ZEEP at T60 (*p* = 0.0453) and compared to PEEP_maxCrs−2_ at T60 (*p* = 0.0202), T120 (*p* = 0.0453), and T180 (*p* = 0.0453). Dopamine was administered in one cat from 4 to 18 min of ventilation with PEEP_maxCrs_ [total dose 0.07 mg/kg (rate of 5 mcg/kg/min)], and in three cats during all timepoints with PEEP_maxCrs+2_ [total dose 0.84 mg/kg (rate of 5 mcg/kg/min), total dose 1.17 mg/kg (rate of 5–10 mcg/kg/min), and total dose 0.46 mg/kg (rate of 5 mcg/kg/min)]. One cat in ZEEP (T30), PEEP_maxCrs_ (T5 and T30), and PEEP_maxCrs+2_ (T5, T30, T60, and T120) had a MAP <60 mmHg either due to insufficient time for dopamine to exert its effect immediately after the ARM or because of the decrease in MAP caused by an additional dose of rocuronium administered close to the time point.

**Table 3:** Selected cardiovascular variables in six (unless indicated within parenthesis) isoflurane-anesthetized cats mechanically ventilated for 3 h with a tidal volume of 10 ml/kg and four end-expiratory pressure (EEP) treatments: zero end-expiratory pressure (ZEEP), positive end-expiratory pressure (PEEP) of highest respiratory system compliance (PEEP_maxCrs_), PEEP_maxCrs_ minus 2 cmH_2_O (PEEP_maxCrs−2_), and PEEP_maxCrs_ plus 2 cmH_2_O (PEEP_maxCrs+2_).

**Variable**	**Group**	**T5**	**T30**	**T60**	**T120**	**T180**
CI	ZEEP	0.23 ± 0.08	0.24 ± 0.08	0.25 ± 0.07	0.26 ± 0.07	0.28 ± 0.06
(L/minute/BW^0.67^)	PEEP_maxCrs−2_	0.22 ± 0.05	0.23 ± 0.06	0.24 ± 0.06	0.25 ± 0.10	0.26 ± 0.08
	PEEP_maxCrs_	0.22 ± 0.02	0.21 ± 0.04	0.23 ± 0.04	0.23 ± 0.04	0.25 ± 0.03
	PEEP_maxCrs+2_	0.23 ± 0.04	0.24 ± 0.03	0.22 ± 0.04	0.24 ± 0.03	0.24 ± 0.03
HR	ZEEP	163 (133–181)	171 (139–187)	174 (146–187)	171 (150–193)	176 (150–181)
(beats/min)	PEEP_maxCrs−2_	169 (151–187)	169 (150–187)	170 (146–187)	167 (153–187)	160(143–193)
	PEEP_maxCrs_	152 (142–187)	168 (146–181)	164 (146–193)	165 (152–193)	173 (153–200)
	PEEP_maxCrs+2_	169 (148–240)	191 (146–240)	185 (148–240)	181 (146–230)	184 (150–230)
SI	ZEEP	0.82 ± 0.08	0.80 ± 0.20	0.90 ± 0.20^*^	0.90 ± 0.20	0.90 ± 0.10
(ml/beat/kg)	PEEP_maxCrs−2_	0.75 ± 0.16	0.79 ± 0.17	0.82 ± 0.19	0.87 ± 0.32	0.91 ± 0.26
	PEEP_maxCrs_	0.82 ± 0.08	0.74 ± 0.15	0.79 ± 0.13	0.80 ± 0.13	0.84 ± 0.11
	PEEP_maxCrs+2_	0.80 ± 0.20	0.70 ± 0.20	0.70 ± 0.20	0.80 ± 0.20	0.80 ± 0.20
MAP	ZEEP	71 (67–113)^*^	69 (57–95)	65 (60–89)	71 (60–89)	67 (58–77)
(mmHg)	PEEP_maxCrs−2_	74 (60–77)	67 (65–69)	72 (66–85)	72 (60–98)	69 (60–74)
	PEEP_maxCrs_	68 (58–95)	65 (58–73)	69 (60–78)	73 (63–83)	72 (66–101) (5)
	PEEP_maxCrs+2_	63 (49–69)	64 (57–74)	61 (59–71)	65 (57–69)	70 (60–81)
SVRI	ZEEP	22,502 (18,994–58,659)	18,231 (16,863–41,995)	19,764 (12,221–30,996)	17,112 (13,620–18,961)	16,510 (15,109–35,785)
(dyne/sec/cm^5^/BW^0.67^)	PEEP_maxCrs−2_	21,954 (17,839–4,917)	19,837 (15,212–37,534)	22,086 (15,749–36,567)	22,068 (12,395–38,281)	18,934 (14,280–36,795)
	PEEP_maxCrs_	22,764 (16,084–29,039)	20,972 (13,082–31,041)	20849 (18,709–27,667)	20,116 (19,403–27,664)	22,676 (16,596–27,934) (5)
	PEEP_maxCrs+2_	19,630 (10,991–26,314)	19,136 (14,533–23,400)	20,903 (14,940–28,519)	19,412 (14,531–26,346)	20,761 (17,182–26,492)
MPAP	ZEEP	13 ± 2^*^	14 ± 2^*^	13 ± 2^*^	13 ± 2^*^	14 ± 3^*^
(mmHg)	PEEP_maxCrs−2_	15 ± 3	16± 3	16 ± 3^*^	16 ± 3^*^	16 ± 3^*^
	PEEP_maxCrs_	16 ± 1	16 ± 1	16 ± 1^*^	17 ± 1	19 ± 1
	PEEP_maxCrs+2_	17 ± 3	19 ± 4	20 ± 3	20 ± 4	20 ± 3
CVP	ZEEP	5 (4–7)^*^	6 (4–9)^*^	6 (4–8)	5 (4–8)	5 (4–10)
(mmHg)	PEEP_maxCrs−2_	7 (4–8)	7 (4–9)	8 (6–8)	8 (4–8)	7 (5–8)
	PEEP_maxCrs_	8 (6–8)	8 (6–10)	8 (7–9)	8 (6–10)	8 (5–10)
	PEEP_maxCrs+2_	8 (6–12)	9 (7–12)	8 (6–11)	8 (6–11)	8 (8–11)
PAOP	ZEEP	8 (7–11)	9 (7–11)	8 (7–11)	8 (6–13)	9 (6–11)
(mmHg)	PEEP_maxCrs−2_	11 (9–14)	12 (9–15)	11 (9–15)	11 (9–15)	10 (8–15)
	PEEP_maxCrs_	10 (7–11)	10 (8–11)	10 (7–11)	10 (9–12)	11 (9–13)
	PEEP_maxCrs+2_	11 (10–12)	10 (9–13)	11 (9–13)	13 (8–13)	11 (9–13)
PVRI	ZEEP	1,709 (1,111–2,251)	1,536 (1,212–1,872)^†^	1,659 (1,111–2,080)^*^	1,436 (865–2,133)^†^	1,605 (842–2,522)^†^
(dyne/sec/cm^5^/m^2^)	PEEP_maxCrs−2_	1,481 (597–2,135)	1,379 (935–1,951)^†^	1,225 (756–2,155)^*^	1,307 (541–2,541)^*^	1,782 (513–2,394)^^*^^
	PEEP_maxCrs_	2,122 (1,587–2,871)	2,310 (1,751–4,233)	2,017 (1,710–4,727)	2,461 (1471–3,438)	2,582 (1,726–3,607)
	PEEP_maxCrs+2_	2,138 (418–4,094)	3,207 (380–3,913)	3,825 (1,339–4,270)	3,306 (753–4,069)	3,026 (997–4,382)

*BW, body weight in kg; CI, cardiac index; HR, heart rate; SI, stroke index; MAP, mean arterial pressure; MPAP, mean pulmonary artery pressure; PAOP, pulmonary artery occlusion pressure; SVRI, systemic vascular resistance index; PVRI, pulmonary vascular resistance index. Data are presented as mean ± standard deviation or median (range)*.

* *Significantly different from PEEP_maxCrs+2_*.

† *Significantly different from PEEP_maxCrs_*.

*T5, T30, T60, T120, and T180 = 5, 30, 60, 120, and 180 min of mechanical ventilation on each specific EEP level, respectively*.

Pulmonary gas exchange and oxygenation variables as well as arterial hemoglobin (Hba) and lactate concentrations are presented in Table 4. DO_2_I was significantly greater at T180 compared to T5 in the cats ventilated with PEEP_maxCrs_ (*p* = 0.05). Ventilation with ZEEP resulted in a lower PaO_2_/FIO_2_ compared to PEEP_maxCrs−2_ (T120, *p* = 0.0121 and T180, *p* = 0.023), PEEP_maxCrs_ (T30, *p* = 0.0269 and T120, *p* = 0.0269), and PEEP_maxCrs+2_ (T30, T60 and T120, *p* = 0.006; T180 *p* = 0.001).

**Table 4:** Pulmonary gas exchange, arterial hemoglobin (Hba) and lactate concentrations, and oxygenation variables in six (unless indicated within parenthesis) isoflurane-anesthetized cats mechanically ventilated for 3 h with a tidal volume of 10 ml/kg and four end-expiratory pressure treatments: zero end-expiratory pressure (ZEEP), positive end-expiratory pressure (PEEP) of highest respiratory system compliance (PEEP_maxCrs_), PEEP_maxCrs_ minus 2 cmH_2_O (PEEP_maxCrs−2_), and PEEP_maxCrs_ plus 2 cmH_2_O (PEEP_maxCrs+2_).

**Variable**	**Group**	**T5**	**T60**	**T120**	**T180**	**T240**
PaO_2_	ZEEP	544 ± 22	530 ± 25	528 ± 16	534 ± 28	522 ± 23
(mmHg)	PEEP_maxCrs−2_	518 ± 34	507 ± 21	513 ± 23	521 ± 17	517 ± 32
	PEEP_maxCrs_	519 ± 30	520 ± 17	509 ± 36	505 ± 40	522 ± 51(5)
	PEEP_maxCrs+2_	519 ± 27	519 ± 17	517 ± 13	529 ± 21	526 ± 38
Hba	ZEEP	9.9 ± 1.1	9.0 ± 0.9	9.0 ± 0.6	9.1 ± 0.7	9.3 ± 0.6
(mg/dl)	PEEP_maxCrs−2_	10.0 ± 1.0	9.4 ± 0.9	9.5 ± 0.9	10.1 ± 0.7	10.2 ± 0.7
	PEEP_maxCrs_	9.9 ± 1.1	9.6 ± 1.1	9.7 ± 1.1	9.9 ± 1.0	10.5 ± 1.2 (5)
	PEEP_maxCrs+2_	10.0 ± 1.1	10.0 ± 0.7	10.2 ± 0.7	10.3 ± 0.7	10.6 ± 0.9
CaO_2_	ZEEP	14.4 (13.8–17.5)	13.3 (12.6–15.3)	13.6 (12.4–14.8)	14.2 (12.6–14.7)	14.0 (12.9–15.2)
(ml/dl)	PEEP_maxCrs−2_	15.2 (12.6–16.2)	14.4 (11.6–15.0)	14.7 (11.9–14.9)	15.3 (13.6–15.7)	15.4 (13.9–16.0)
	PEEP_maxCrs_	14.6 (13.1–17.2)	14.0 (13.0–16.8)	13.8 (13.6–17.2)	14.7 (13.1–17.0)	15.8 (13.9–17.9) (5)
	PEEP_maxCrs+2_	14.5 (13.6–17.4)	15.1 (13.8–16.3)	15.2 (13.9–16.7)	15.4 (14.0–16.7)	15.5 (14.2–17.9)
S$\overset{\bar{}}{v}$O_2_ (%)	ZEEP	79.7 ± 8.2	78.4 ± 8.2	79.7 ± 7.5	80.4 ± 6.6	81.9 ± 5.7
	PEEP_maxCrs−2_	80.6 ± 5.4	80.8 ± 4.9	80.9 ± 5.3	81.9 ± 4.3	82.1 ± 3.1
	PEEP_maxCrs_	76.1 ± 5.8	74.0 ± 5.7	74.8 ± 5.2	78.4 ± 5.9	78.2 ± 6.3
	PEEP_maxCrs+2_	73.9 ± 9.5	79.7 ± 3.5	79.5 ± 5.0	78.9 ± 3.7	78.7 ± 4.9
DO_2_I	ZEEP	34.6 ± 11.8	32.9 ± 10.3	34.7 ± 11.2	36.1 ± 10.6	38.8 ± 9.3
(ml/minute)	PEEP_maxCrs−2_	32.4 ± 8.2	32.6 ± 9.4	34.5 ± 10.2	38.6 ± 16.3	38.3 ± 13.5
	PEEP_maxCrs_	32.1 ± 4.0	30.3 ± 4.5	32.6 ± 4.9	34.4 ± 5.2	38.9 ± 6.0^#^ (5)
	PEEP_maxCrs+2_	33.7 ± 6.5	35.4 ± 5.6	33.4 ± 7.1	36.3 ± 5.9	38.1 ± 7.3
VO_2_I	ZEEP	10.2 ± 2.7	9.7 ± 2.8	9.6 ± 2.5	10.5 ± 2.2	10.5 ± 2.3
(ml/minute)	PEEP_maxCrs−2_	8.5 ± 2.7	8.0 ± 2.6	8.9 ± 2.3	8.8 ± 3.6	9.0 ± 2.6
	PEEP_maxCrs_	9.6 ± 0.9	9.9 ± 1.1	10.4 ± 1.2	9.4 ± 2.1	10.6 ± 1.1 (5)
	PEEP_maxCrs+2_	10.9 ± 4.0	9.8 ± 1.7	8.9 ± 4.1	10.0 ± 2.2	10.0 ± 2.4
O_2_ER	ZEEP	0.31 ± 0.07	0.31 ± 0.10	0.29 ± 0.08	0.30 ± 0.06	0.28 ± 0.05
	PEEP_maxCrs−2_	0.26 ± 0.06	0.25 ± 0.06	0.27 ± 0.07	0.23 ± 0.05	0.23 ± 0.02^#^
	PEEP_maxCrs_	0.30 ± 0.06	0.33 ± 0.05	0.32 ± 0.06	0.28 ± 0.07	0.28 ± 0.05 (5)
	PEEP_maxCrs+2_	0.32 ± 0.10	0.28 ± 0.04	0.26 ± 0.07	0.28 ± 0.04	0.27 ± 0.05
Qs/Qt (%)	ZEEP	4.3 ± 3.4	4.9 ± 3.2	4.7 ± 2.0	3.7 ± 1.3	4.9 ± 1.4
	PEEP_maxCrs−2_	5.4 ± 3.7	6.5 ± 3.3	5.4 ± 1.3	5.3 ± 2.0	5.3 ± 2.5
	PEEP_maxCrs_	4.4 ± 1.6	4.1 ± 1.1	4.7 ± 2.2	5.8 ± 3.4	4.1 ± 3.2 (5)
	PEEP_maxCrs+2_	4.1 ± 0.6	4.4 ± 1.0	5.0 ± 1.3	3.8 ± 1.2	4.4 ± 3.6
PaO_2_/FIO_2_	ZEEP	572 ± 20	558 ± 25^*^^†^	558 ± 20^*^	561 ± 26^*^^†^	548 ± 22^*^
(mmHg)	PEEP_maxCrs−2_	585 ± 38	573 ± 36	580 ± 23	589 ± 15	584 ± 38
	PEEP_maxCrs_	583 ± 33	586 ± 19	575 ± 41	589 ± 12	588 ± 59 (5)
	PEEP_maxCrs+2_	582 ± 28	586 ± 19	584 ± 17	598 ± 22	593 ± 47
P(a-ET)CO_2_	ZEEP	2.5 ± 0.9	3.1 ± 1.8	3.7 ± 1.6	4.4 ± 2.1	4.6 ± 1.9
(mmHg)	PEEP_maxCrs−2_	3.3 ± 1.2	3.9 ± 1.3	3.6 ± 1.2	3.6 ± 1.4	4.0 ± 1.8
	PEEP_maxCrs_	2.9 ± 0.9	2.8 ± 1.4	2.2 ± 1.5	3.0 ± 0.9	3.4 ± 2.5 (5)
	PEEP_maxCrs+2_	3.2 ± 1.4	3.4 ± 1.4	3.1 ± 1.0	3.1 ± 1.2	2.8 ± 1.6
Lactate (mmol/L)	ZEEP	1.6 (1.1–3.5)	1.5 (1.0–3.0)	1.4 (0.9–3.0)	1.7 (1.0–3.1)	1.8 (1.0–3.2)
	PEEP_maxCrs−2_	1.6 (1.1–3.6)	1.6 (1.1–3.2)	1.6 (1.0–3.3)	1.7 (1.2–3.3)	1.7 (1.2–3.4)
	PEEP_maxCrs_	2.0 (1.0–4.4)	1.9 (1.1–3.7)	2.0 (1.2–3.8)	2.0 (1.5–4.9)	1.9 (1.3–4.0) (5)
	PEEP_maxCrs+2_	1.8 (1.4–4.1)	1.7 (1.3–3.5)	1.7 (1.4–3.6)	1.6 (1.1–3.9)	1.8 (1.2–3.8)

*Data are presented as mean ± standard deviation or median (range)*.

*PaO_2_, arterial partial pressure of oxygen; CaO_2_, arterial blood oxygen content; S$\overset{\bar{}}{v}$O_2_, mixed venous blood oxygen hemoglobin saturation; DO_2_I, oxygen delivery index; VO_2_I, oxygen consumption index; O_2_ER, oxygen extraction ratio; Qs/Qt, shunt fraction; P(a-ET)CO_2_, arterial to end-tidal CO_2_ partial pressure gradient; PaO_2_/FIO_2_, ratio between PaO_2_ and inspiratory fraction of oxygen*.

* *Significantly different from PEEP_maxCrs+2_*.

† *Significantly different from PEEP_maxCrs_*.

*Significantly different than PEEP_maxCrs−2_*.

*T5, T30, T60, T120, and T180 = 5, 30, 60, 120, and 180 min of mechanical ventilation on each specific EEP level, respectively*.

The acid–base variables are presented in Table 5. The cats ventilated with ZEEP had a lower PaCO_2_ compared to PEEP_maxCrs+2_ at T5 (*p* = 0.0349) and T30 (*p* = 0.0127), and to PEEP_maxCrs_ at T5 (*p* = 0.0432).

**Table 5:** Acid-base variables in six (unless indicated within parenthesis) isoflurane-anesthetized cats mechanically ventilated for 3 h with a tidal volume of 10 ml/kg and four end-expiratory pressure (EEP) treatments: zero end-expiratory pressure (ZEEP), positive end-expiratory pressure (PEEP) of highest respiratory system compliance (PEEP_maxCrs_), PEEP_maxCrs_ minus 2 cmH_2_O (PEEP_maxCrs−2_), and PEEP_maxCrs_ plus 2 cmH_2_O (PEEP_maxCrs+2_).

**Variable**	**Group**	**T5**	**T30**	**T60**	**T120**	**T180**
pH	ZEEP	7.396 ± 0.034	7.386 ± 0.042	7.378 ± 0.036	7.381 ± 0.026	7.377 ± 0.038
	PEEP_maxCrs−2_	7.368 ± 0.036	7.356 ± 0.032	7.356 ± 0.039	7.341 ± 0.030	7.340 ± 0.038
	PEEP_maxCrs_	7.360 ± 0.052	7.359 ± 0.046	7.371 ± 0.043	7.358 ± 0.038	7.346 ± 0.029
	PEEP_maxCrs+2_	7.367 ± 0.027	7.348 ± 0.029	7.355 ± 0.031	7.361 ± 0.032	7.349 ± 0.028
PaCO_2_	ZEEP	31.3 ± 1.6^*^^†^	32.9 ± 2.7^*^	33.9 ± 2.3	34.1 ± 2.1	34.8 ± 3.5
(mmHg)	PEEP_maxCrs−2_	34.1 ± 2.4	35.4 ± 2.9	35.3 ± 3.5	36.1 ± 2.2	36.3 ± 3.0
	PEEP_maxCrs_	35.1 ± 2.4	35.6 ± 2.4	34.0 ± 1.7	34.9 ± 1.2	36.3 ± 2.1
	PEEP_maxCrs+2_	35.2 ± 2.7	37.3 ± 2.2	36.2 ± 2.8	35.4 ± 2.0	36.0 ± 2.0
BE	ZEEP	−5.2 ± 1.5	−5.0 ± 1.6	−4.9 ± 1.5	−4.6 ± 1.4	−4.4 ± 1.3
(mmol/L)	PEEP_maxCrs−2_	−5.4 ± 0.9	−5.4 ± 0.9	−5.4 ± 0.9	−5.8 ± 1.3	−5.8 ± 1.1
	PEEP_maxCrs_	−5.2 ± 2.0	−5.0 ± 1.8	−5.2 ± 1.7	−5.4 ± 2.0	−5.3 ± 2.1
	PEEP_maxCrs+2_	−4.8 ± 1.1	−4.9 ± 1.1	−5.0 ± 0.6	−5.0 ± 0.9	−5.4 ± 1.2

*PaCO_2_, arterial partial pressure of carbon dioxide; BE, base excess*.

*Data are presented as mean ± standard deviation*.

* *Significantly different from PEEP_maxCrs+2_*.

† *Significantly different from PEEP_maxCrs_*.

*T5, T30, T60, T120, and T180 = 5, 30, 60, 120, and 180 min of mechanical ventilation on each specific EEP level, respectively*.

## Discussion

Mechanical ventilation is commonly used during the anesthetic management of cats (22). However, the effects of different ventilatory settings on gas exchange and the cardiovascular system are poorly understood in this species. The present study aimed to partially fill this knowledge gap by investigating the effects on the cardiovascular system, and on gas exchange and arterial oxygenation of four different levels of EEP during 3 h of mechanical ventilation, which encompasses the duration of most anesthetic procedures performed in cats. The main findings of this study were that (1) all levels of PEEP studied minimally improved arterial oxygenation with no significant improvement in DO_2_I when compared to ZEEP; (2) PEEP_maxCrs_ and PEEP_maxCrs+2_ were associated with lower MAP and higher requirements for dopamine to maintain MAP > 60 mmHg compared to ZEEP and PEEP_maxCrs−2_; and (3) PEEP_maxCrs_ and PEEP_maxCrs+2_ resulted in higher MPAP and PVRI than ZEEP and PEEP_maxCrs−2_.

The ideal PEEP to be used in anesthetized patients has been a topic of debate. A fixed PEEP of 5 cmH_2_O after an ARM has been shown to prevent atelectasis and improve arterial oxygenation in dogs (3). However, variables commonly found in clinical cases such as obesity, surgical procedure (e.g., laparoscopic surgeries), and concurrent lung disease may alter the PEEP that can provide optimal improvement in pulmonary function. A recent guideline on lung-protective ventilation for surgical human patients (23) suggested that individualized mechanical ventilation settings including PEEP can improve clinical outcomes (24). One of the first methods described to individualize PEEP used the PEEP_maxCrs_ achieved during a decremental PEEP titration, which was associated with optimal cardiopulmonary function in critical human patients (25). In addition, PEEP_maxCrs_ promoted a better balance between preventing alveolar tidal recruitment/derecruitment and overdistention when compared to higher PEEP or ZEEP in lung-healthy rats (11). This beneficial effect of PEEP_maxCrs_ may explain why this level of PEEP was associated with improved clinical outcomes in critical human patients (10). Levels of PEEP higher and lower than PEEP_maxCrs_ such as PEEP_maxCrs−2_ and PEEP_maxCrs+2_ have been investigated to better understand the balance between the improvement in pulmonary function and the possible detrimental effects of PEEP, such as decrease in MAP and CI (5, 26). Although most clinical studies using PEEP_maxCrs_ have been performed in critical patients, healthy cats can develop atelectasis during anesthesia (6), which can predispose to ventilator-induced lung injury. Therefore, the results reported in this study may significantly contribute to the management of mechanical ventilation in lean healthy cats, as well as serve as reference for future studies in this species using PEEP in a variety of clinical conditions, including obesity and critical illness.

### Cardiovascular Effects

Despite its beneficial effects on pulmonary function, PEEP can significantly decrease CI and MAP, as recently demonstrated in dogs, especially when PEEP was higher than PEEP_maxCrs_ (5). Similar effects were found in cats, with PEEP_maxCrs+2_ causing a more sustained and significant decrease in cardiovascular function as illustrated by the lower MAP at T5 when compared to ZEEP and by the need for dopamine to maintain MAP > 60 mmHg during the entire ventilation period in 3 out of 6 cats. As observed in dogs (5, 27, 28), high PEEP decreased SI, an important contributing factor for the more significant cardiovascular depression during PEEP_maxCrs+2_ in the cats of this study. The administration of dopamine to maintain MAP > 60 mmHg masked the magnitude of the actual depression in SI, CI, and MAP caused by PEEP, particularly at PEEP_maxCrs+2_. Dopamine was used in this experiment because it was considered unethical to tolerate severe hypotension in a survival study. Although PEEP decreases CI and MAP mainly by decreasing venous return with no apparent decrease in ventricular function (27), dopamine, at predominantly positive inotropic doses, is effective to treat the decrease in CI and MAP caused by PEEP in human patients with acute respiratory failure (29). At the doses used in the present study, dopamine has a predominant positive inotropic effect in cats (30, 31). However, selective venoconstriction caused by low doses of dopamine (32) promoting an increase in venous return is another possible mechanism for the improvement in CI and MAP in the cats ventilated with PEEP_maxCrs_ and PEEP_maxCrs+2_. At the doses used in this study (5 and 10 mcg/kg/min), dopamine was effective at maintaining CI and MAP at values similar to spontaneously breathing cats anesthetized with comparable ET_ISO_ (33) and can be considered a good option to manage the cardiovascular depression caused by PEEP in cats.

Only one cat ventilated with PEEP_maxCrs_ required dopamine during the first 20 min of the ventilation protocol. This effect was likely related to the summation of cardiovascular depression caused by the ARM performed immediately before the ventilation protocol and the PEEP settings and has been reported in dogs (5). We attempted to minimize the influence of the cardiovascular depression caused by the ARM by administering a bolus of isotonic crystalloids in all cats immediately before the ARM, as described in dogs (34). A temporal improvement in CI due to fluid retention related to sustained positive pressure ventilation with PEEP (35) and/or a possible temporal decrease in the cardiovascular depression of isoflurane (36) could have also played a role in the lack of need for dopamine in this cat after 20 min of ventilation. However, both mechanisms of a temporal increase in CI take more than 20 min to occur (35, 36) and were deemed unlikely to have contributed. Interestingly, a temporal increase in CI was observed in dogs after 180 min of ventilation at different levels of EEP (5). Although a tendency (*p* = 0.0629) of higher CI was noted at T180 when compared to T5 with all studied EEPs, this effect was not detected in the present study because of three possible reasons: (1) small sample size and its associated low statistical power, (2) species-specific differences in the dynamics of fluid retention and isoflurane effects between dogs and cats, and (3) the confounding factor of the use of dopamine.

The augmented lung volume caused by PEEP can increase MPAP and PVRI (5, 28). In this study, PVRI was higher during ventilation with PEEP_maxCrs_ and PEEP_maxCrs+2_ when compared to ZEEP and PEEP_maxCrs−2_. Therefore, caution should be used when using those levels of PEEP in cats with right ventricular dysfunction and pulmonary hypertension. The increased right ventricle afterload associated with higher PVRI in PEEP_maxCrs_ and PEEP_maxCrs+2_ was a probable contributor to the decrease in CI and MAP at these PEEP levels. In contrast with these findings, PVRI was less affected by PEEP in dogs since it only increased when a PEEP 4 cmH_2_O higher than PEEP_maxCrs_ was used (5). The use of dopamine and lower *V*_*T*_ in the present study are the main methodological differences between the previous dog study and are unlikely to explain the increase in PVRI at lower PEEP levels in cats when compared to dogs. Intravenous dopamine at doses up to 20 mcg/kg/min was not associated with increased PVRI in cats (31). The highest dose used in this study was 10 mcg/kg/min. The lower *V*_*T*_ used in the cats is expected to be associated with a lower PVRI as PVRI increases with *V*_*T*_ (37). This difference can likely be related to a species-specific difference in the effects of PEEP, as data from the same laboratory indicates that PVRI is higher in cats than in dogs (19, 31).

### Gas Exchange

The decreased atelectasis and increased FRC caused by PEEP have been associated with significant improvement in pulmonary gas exchange (1, 3). Nevertheless, the improvement in PaO_2_/FIO_2_ observed in all studied PEEP levels when compared to ZEEP are considered small, with minimal clinical significance since PaO_2_/FIO_2_ remained within normal limits with all treatments. The improvement in PaO_2_/FIO_2_ caused by PEEP was more important toward the end of the ventilation protocol and was likely caused by a faster temporal development of atelectasis and deterioration of respiratory mechanics in the cats ventilated with ZEEP compared to PEEP as previously demonstrated in dogs (3) and rats (11). When excessive, PEEP can cause alveolar overdistention and decrease pulmonary perfusion, which can ultimately impair pulmonary gas exchange as a consequence of increased $\overset{∙}{\text{V}}$/$\overset{∙}{\text{Q}}$ (alveolar dead space) (13). None of the PEEP treatments appeared to increase $\overset{∙}{\text{V}}$/$\overset{∙}{\text{Q}}$ in cats because no increase in P(a-ET)CO_2_ was observed with PEEP even at its highest level. Increased P(a-ET)CO_2_ has been commonly used as a marker of alveolar dead space but it has serious limitations in the presence of increased Qs/Qt, as demonstrated in anesthetized horses (38). Because Qs/Qt was normal in all EEP treatments, it is safe to assume that the lack of elevation in P(a-ET)CO_2_ caused by PEEP in this study indicated no increase in alveolar dead space. Similar effects of PEEP on PaO_2_/FIO_2_ and P(a-ET)CO_2_ were observed in dogs (5) and future studies are necessary to clarify the clinical significance of the mildly improved pulmonary gas exchange promoted by PEEP in healthy cats.

### Oxygen Delivery

One of the clinical goals of improving arterial oxygenation in anesthetized patients is to increase CaO_2_ and consequently DO_2_I. In critically ill human patients, PEEP_maxCrs_ was associated with optimal cardiopulmonary function with improved DO_2_I (25). However, this was not achieved in healthy cats with any of the PEEP treatments. The CI depression caused by PEEP, especially at high levels, can be associated with a detrimental effect on DO_2_I (5). Interestingly, DO_2_I improved at T180 when compared to T5 in the cats ventilated with PEEP_maxCrs_, probably due to a combination of nonsignificant improvements in CI and CaO_2_ over time observed with this level of PEEP. In dogs ventilated with PEEP_maxCrs_ and PEEP_maxCrs+2_, a similar temporal improvement in DO_2_I was caused by a progressive increase in CI at the fourth hour of ventilation. There was a tendency of a temporal increase in DO_2_I at the other levels of PEEP and is possible that the small sample size used in this study did not provide enough power to reach statistical differences.

### Limitations

This study has important limitations that need to be accounted for when interpreting the results. As previously discussed, the use of dopamine to treat hypotension during the experiments has mitigated the decrease in CI and MAP due to PEEP_maxCrs_ and PEEP_maxCrs+2_. However, the dose of dopamine required to maintain MAP > 60 mmHg provides an indirect but reliable assessment of the cardiovascular depression related to each treatment. In cats, ventilation with lower FIO_2_ (0.4) improved lung aeration distribution with less atelectasis and better gas exchange than at FIO_2_ close to 1.0 (6). Therefore, it is possible that the beneficial effect of PEEP on gas exchange observed in this study would not be achieved if the cats were ventilated with a lower FIO_2_ as previously reported in dogs (3). The results reported on healthy lean cats in dorsal recumbency should be taken with caution when applied to different body positions (e.g., lateral or sternal recumbency), cats with a different body condition score and critically ill cats, and especially cats with pulmonary disease because all these conditions are associated with altered respiratory mechanics and gas exchange where PEEP_maxCrs_ could be different than the conditions in this study. The administration of 10 ml/kg of lactated Ringer's solution before an ARM has been shown to minimize the decrease in MAP and CI in dogs (34) before the ARM. The same technique probably made the cats of the present study less sensitive to the preload effects of PEEP, and because of that, the cardiovascular effects of PEEP will likely be magnified in cats not receiving the same fluid bolus. At the first 30 min of ventilation, PaCO_2_ was higher in the cats ventilated with PEEP_maxCrs_ and PEEP_maxCrs+2_ when compared to ZEEP and was probably an incidental finding due to the initial adjustments of RR after the ARM. These PaCO_2_ differences could have promoted an increase in sympathetic tone improving MAP and CI (39), masking further depression of CI and MAP by those PEEP values. This effect was unlikely to have affected our results since significant improvements of CI and MAP were only observed with differences of PaCO_2_ higher than approximately 17 mmHg (39). Other confounding factors such as ET_ISO_ and body temperature were well controlled during the experiments with no difference between treatments or time points. Finally, the cats of this study did not undergo any surgical procedure, which can produce significant changes in cardiovascular function, respiratory mechanics, and gas exchange, especially with intraabdominal or intrathoracic procedures. For instance, laparotomy and thoracotomy caused significant changes in lung compliance and resistance in rats (40, 41), which can lead to different requirements of PEEP to achieve optimal cardiopulmonary function.

### Conclusions

In isoflurane-anesthetized lung-healthy cats ventilated for 3 h with a *V*_*T*_ of 10 ml/kg after an ARM:

- none of the PEEP levels studied promoted clinically significant improvement in gas exchange;
- PEEP_maxCrs_ and PEEP_maxCrs+2_ produced more cardiovascular depression, which was mild and limited to the first 20 min of ventilation in PEEP_maxCrs_;
- none of the PEEP levels improved DO_2_I but a temporal increase on this variable was observed, particularly with PEEP_maxCrs_;
- the cardiovascular effects of PEEP_maxCrs−2_ were not significantly different than ZEEP;
- dopamine was effective at mitigating the cardiovascular depression produced by PEEP; and
- the effects of these levels of EEP on respiratory mechanics and ventilation-induced lung injury, as well as their use in different clinical situations, such as obese and critically ill cats, deserve future investigation.

## Data Availability Statement

The raw data supporting the conclusions of this article will be made available by the authors, without undue reservation.

## Ethics Statement

The animal study was reviewed and approved by University of California Davis Institutional Animal Use and Care Committee.

## Author Contributions

MM: study execution, data acquisition, analysis, interpretation, and preparation of the manuscript, approved the final manuscript, and full access to all the data and responsible for integrity of the data and accuracy of data analysis. JS: study design, study execution, data acquisition, analysis, interpretation, and preparation of the manuscript, approved the final manuscript, and full access to all the data and responsible for integrity of the data and accuracy of data analysis. BP: study execution, data analysis, interpretation and preparation of the manuscript, and approved the final manuscript. AA: study execution, data acquisition, analysis, interpretation, and preparation of the manuscript, and approved the final manuscript. GM-R, FJ, and CB: data analysis, interpretation, and preparation of the manuscript and approved the final manuscript. All authors contributed to the article and approved the submitted version.

## Funding

This work was supported by Center for Companion Animal Health (Resident Grant No. 38665 and Faculty Grant No. 44336), School of Veterinary Medicine, University of California, Davis.

## Conflict of Interest

The authors declare that the research was conducted in the absence of any commercial or financial relationships that could be construed as a potential conflict of interest.

## Publisher's Note

All claims expressed in this article are solely those of the authors and do not necessarily represent those of their affiliated organizations, or those of the publisher, the editors and the reviewers. Any product that may be evaluated in this article, or claim that may be made by its manufacturer, is not guaranteed or endorsed by the publisher.
